# lncRNA CDKN2B-AS1 Could Be an Indicator to Identify Prognosis and Status of Immune Microenvironment in Thyroid Cancer

**DOI:** 10.1155/2022/4317480

**Published:** 2022-04-08

**Authors:** Cheng Xue, Cheng Yi, Haiyang Lin, Jiajia Zhao, Junhui Yuan, Yongzheng Chen, Haibing Chen, Li Lin, Yisha Zhao

**Affiliations:** ^1^Department of Endocrinology, Wenling First People's Hospital, Wenling, China; ^2^Department of Pediatrics, Wenling Maternal and Child Health Care Hospital, Wenling, China

## Abstract

Thyroid cancer (TC) is prone to recurrence, and biomarkers for predicting progression-free interval (PFI) are poorly explored. The study investigates the predictive value and underlying biological mechanisms of lncRNA CDKN2B-AS1 in TC. Combining RNA-seq and survival data, we identified that CDKN2B-AS1 was upregulated in TC samples and could be an excellent prognostic indicator. To exclude confounding factors, we divided patients into different subgroups using median CDKN2B-AS1 expression, and the effects of CDKN2B-AS1 on PFI and clinical features were explored in different clinical subgroups. Meanwhile, ssGSEA and ESTIMATE algorithms revealed that CDKN2B-AS1 overexpression may suggest the active state of the immune microenvironment. On the other hand, enrichment analysis also proved the potential influence of CDKN2B-AS1 on immune regulation in TC. Finally, we created a CDKN2B-AS1, microRNAs, and TFs network and discovered a new biomarker (CDKN2B-AS1) that might be employed as a therapeutic target in TC patients.

## 1. Introduction

The incidence of thyroid cancer (TC) is increasing [1]. Although treatment in TC has been developed rapidly in recent years, specific pathogenesis is still unclear [2]. The occurrence of TC is a complex process involving multiple pathways, and molecular mechanism in TC has been a hot topic for researchers. lncRNAs were initially thought to be transcriptional noise of the genome without biological function [3], but recent studies have confirmed that lncRNAs play an essential role in the development of tumors [4]. Meanwhile, tumor-infiltrating immune cells (TIIC), as part of the tumor microenvironment (TME), demonstrated that lncRNA mediates communication between it and tumor cells [5]. However, only a few lncRNA effects on microenvironment and prognosis in TC have been adequately explained.

CDKN2B-AS1 has been implicated in several cancers as an oncogene. CDKN2B-AS1 is located in the CDKN2B-CDKN2A gene cluster on chromosome 9p21. CDKN2B-AS1 induces renal clear cell carcinoma by recruiting CREB-binding proteins and three epigenetically modified complexes comprising SET and MYND structural domains to the NUF2 promoter region [6]. In addition, CDKN2B-AS1 works as a sponge in lung cancer, adsorbing miR-378b and regulating miR-378b/NR2C2 [7]. However, so far, few studies have investigated how CDKN2B-AS1 plays a role in TC.

Through bioinformatics algorithms, the purpose of our study was to deduce the critical function of CDKN2B-AS1 in TC. The TCGA database was utilized to validate CDKN2B-AS1 differential expression and clinical significance in TC and pancancers. The impact of various CDKN2B-AS1 expression levels on immune cell content was assessed using the ssGSEA algorithm. Meanwhile, three types of enrichment analysis approaches were conducted to determine the precise pathways affecting the immune microenvironment in TC tissues when CDKN2B-AS1 was overexpressed. Finally, regulatory networks based on CDKN2B-AS1 were built for future in vitro studies.

## 2. Materials and Methods

### 2.1. Datasets and Preliminary Screening

A total of 502 TC samples and 58 normal samples were included in this study. RNA-seq (FPKM) and clinical data were obtained from TCGA databases [8]. To identify potential protooncogenes in TC, we performed a preliminary screen among the 14077 lncRNAs annotated. Limma package was used to screen out the differential expression lncRNAs list (adj. *P* value < 0.05, ∣log FC | >1). The univariate Cox regression analysis was performed on all lncRNAs with disease progression as the dependent variable to identify potential risk factors (*P* value < 0.05, HR > 1) [9]. The lncRNAs identified by the above two methods were overlapped to obtain candidate lncRNAs. Finally, redundant lncRNAs were removed using LASSO regression analysis to obtain CDKN2B-AS1 [10].

### 2.2. Clinical Correlation Analysis

We obtained the CDKN2B-AS1 expression of each patient for determining the median value, which is used to select high-CDKN2B-AS1 and low-CDKN2B-AS1 groups. The Kaplan-Meier analysis was performed to compare differences in PFI between different clinical subgroups [11]. The Wilcoxon rank sum test was used to explore difference of clinical features and CDKN2B-AS1 expression. T stage, N stage, M stage, clinical stage, lymphovascular invasion, age, and gender are among the clinical characteristics examined in this study. We also created a nomogram based on CDKN2B-AS1 expression using the rms and survival programs.

### 2.3. Enrichment Analysis

Kyoto Encyclopedia of Genes and Genomes [12] and Gene Ontology analysis was used to explore the possible biological processes (clusterProfiler package for performing enrichment and (

http://org.hs.eg.)db package for transferring symbol ID). In addition, we conducted gene set enrichment analysis (GSEA) based on hallmarks geneset from MSigDB Collections (false discovery rate (FDR) < 0.25 and *p* adjust < 0.05) [13].

### 2.4. Immune-Infiltration Analysis

The content of 24 immune cells in TC was determined using the ssGSEA algorithm. In addition, the stromal score, immune score, and estimate score were analyzed using the ESTIMATE algorithm, and the purpose of the above scores was to quantify TME in TC. *T* test was used to compare the difference of immune cell content and TME score in different CDKN2B-AS1 expression groups [14].

### 2.5. Targeting Network

We used comprehensive gene expression profiling and network visual analytics (NetworkAnalyst) to visualize the targeting network [15]. The literature-curated regulatory interaction information was collected from the RegNetwork repository.

### 2.6. Statistical Analysis

All statistical analyses were performed using the R software (v.3.6.3). Detailed statistical methods about transcriptome data processing are covered in the above section.

## 3. Result

### 3.1. Identification of CDKN2B-AS1 in TC Samples

**Figure 1:**
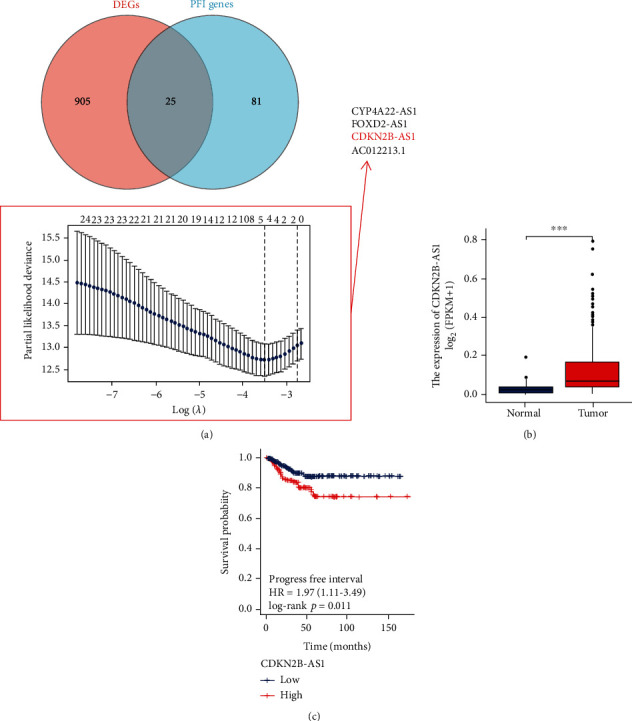
Screening of CDKN2B-AS1 in TC. (a) The overlap of DEGs and PFI genes was followed by the elimination of redundant lncRNAs using LASSO algorithm. (b) CDKN2B-AS1 expression in TCGA-TC cohort. (c) A survival curve of progression-free interval for TC patients. ^∗∗∗^*p* < 0.001.

Firstly, Limma package was used to screen out the differential expression lncRNAs (DELs), and 930 overexpression lncRNAs were obtained. Subsequently, 106 PFI lncRNAs were screened using *P* value < 0.05 and HR > 1 as threshold in the Cox regression analysis. Finally, redundant lncRNAs were removed using LASSO regression analysis (Figure 1(a)). From the four lncRNAs (CYP4A22-AS1, FOXD2-AS1, CDKN2B-AS1, and AC012213.1), we selected CDKN2B-AS1 for subsequent analysis. The subsequent Wilcoxon rank sum test and log-rank test again demonstrated the upregulation of CDKN2B-AS1 in tumor tissues (Figure 1(b)) and its prognostic value (Figure 1(c)).

Taken together, our data show that CDKN2B-AS1 had an abnormal expression in TC tissues.

### 3.2. Correlation between CDKN2B-AS1 Expression and Clinical Characteristics

**Figure 2:**
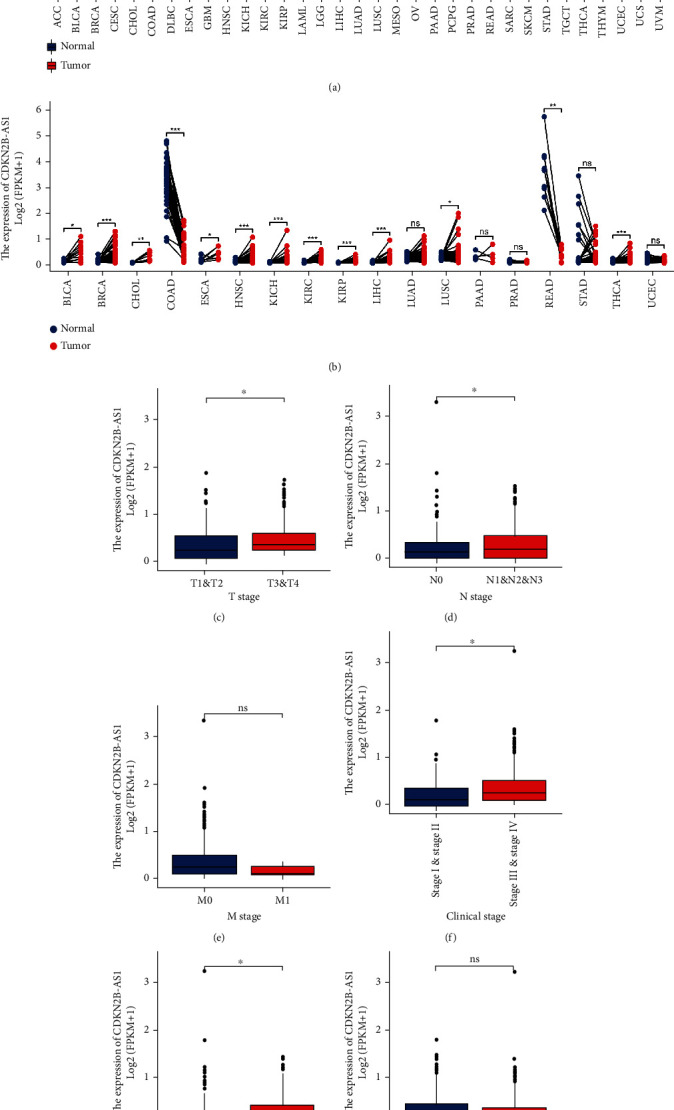
Clinical characteristic analysis. (a) Unpaired differential expression analysis of CDKN2B-AS1 in the TCGA database. (b) Paired differential expression analysis of CDKN2B-AS1 in the TCGA database. Correlation analysis of (c) T stage, (d) N stage, (e) M stage, (f) clinical stage, (g) lymphovascular invasion, and (h) age with CDKN2B-AS1 expression. ^∗^*p* < 0.05.

In a pancancer project of TCGA database, it was found that in unpaired and paired difference analysis, most tumors were CDKN2B-AS1 overexpressed except for COAD and READ paracancerous normal tissues where CDKN2B-AS1 was upregulated (Figures 2(a) and 2(b)). In addition, the expression of CDKN2B-AS1 was upregulated in different subgroups, including T3 and T4 subgroup (Figure 2(c)), N1 and N2 and N3 subgroup (Figure 2(d)), stage III and IV subgroup (Figure 2(f)), and lymphatic invasion subgroup (Figure 2(g)). However, there was no statistical difference in the expression of CDKN2B-AS1 in M stage (Figure 2(e)) and age (Figure 2(h)).

Taken together, our data show that CDKN2B-AS1 could stratify patients with different clinical characteristics.

### 3.3. Survival Analysis in Different Subgroups

To further clarify the effectiveness of CDKN2B-AS1 in indicating disease recurrence in different subgroups, the Kaplan-Meier survival analysis was performed for each clinical subgroup. In stage subgroups, we found that higher expression of CDKN2B-AS1 was associated with shorter PFI time in stage I subgroup (Figure 2(a)), younger subgroup (Figure 2(e)), and female subgroup (Figure 2(g)). In addition, stage II-IV subgroups (Figures 2(b)–2(d)), older subgroup (Figure 2(f)), and male subgroup (Figure 2(h)) also should be noted although CDKN2B-AS1 expression in the above subgroups was not statistically significant.

Taken together, our data show that CDKN2B-AS1 could guide prognosis in TC patients.

### 3.4. CDKN2B-AS1 Regulates Immune-Infiltration in TC Tissues

**Figure 3:**
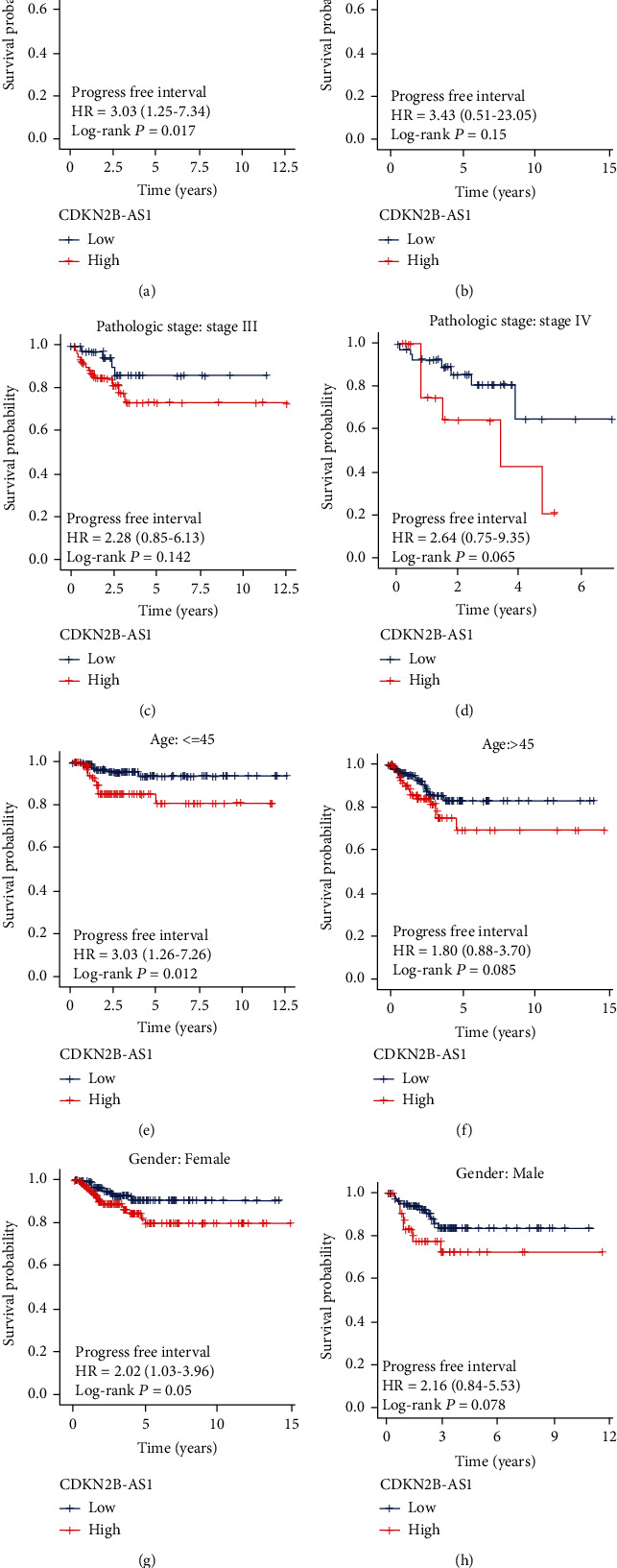
Subgroup survival analysis. Survival analysis of clinical subgroups, including (a) stage I subgroup, (b) stage II subgroup, (c) stage III subgroup, (d) stage IV subgroup, (e) young subgroup, (f) old subgroup, (g) female subgroup, and (h) male subgroup.

Interestingly, we found that most of the immune cells were upregulated in the CDKN2B-AS1 high expression group, except for Th17 cells, Tgd, pDC, and NK cells (Figure 3(a)). In addition, we used ESTIMATE algorithm to quantify TME in TC. Not surprisingly, the CDKN2B-AS1 high expression group had higher stromal score (Figure 3(b)), immune score (Figure 3(c)), and ESTIMATE score (Figure 3(d)) in TC tissues.

### 3.5. Construction of Nomogram

**Figure 4:**
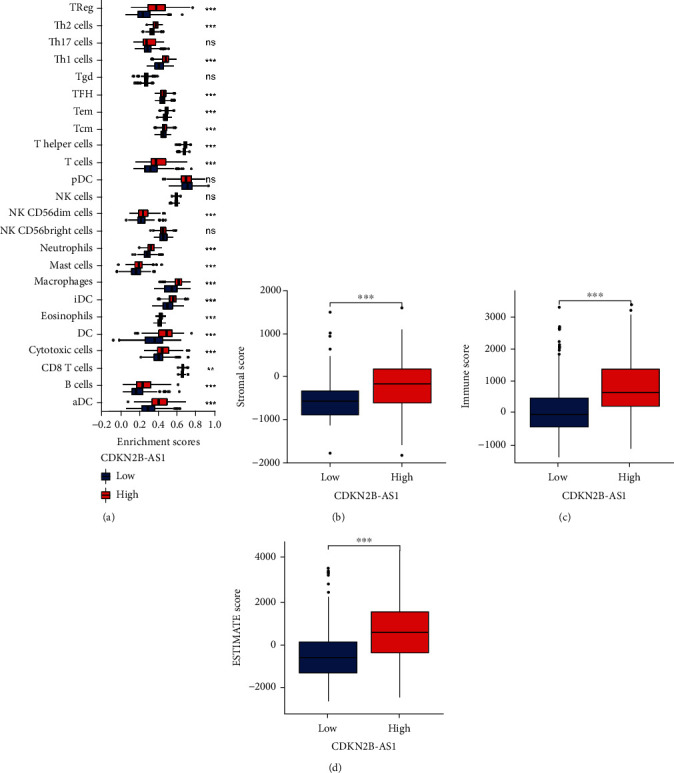
Immune-infiltration analysis. (a) Differential analysis of 24 immune cells. Differential analysis of ESTIMATE algorithm, including (b) stromal score, (c) immune score, and (d) ESTIMATE score. ^∗^*p* < 0.05, ^∗∗^*p* < 0.01, and ^∗∗∗^*p* < 0.001.

To facilitate clinical application, we combined CDKN2B-AS1 expression and pathological stage to construct a nomogram that can predict disease recurrence (Figure 4(a)). The calibration curve in the TCGA-TC cohort illustrated that the nomogram has good predictive significance and our prediction curve was close to the standard curve (Figures 4(b)–4(d)).

### 3.6. Potential Mechanisms of CDKN2B-AS1

**Figure 5:**
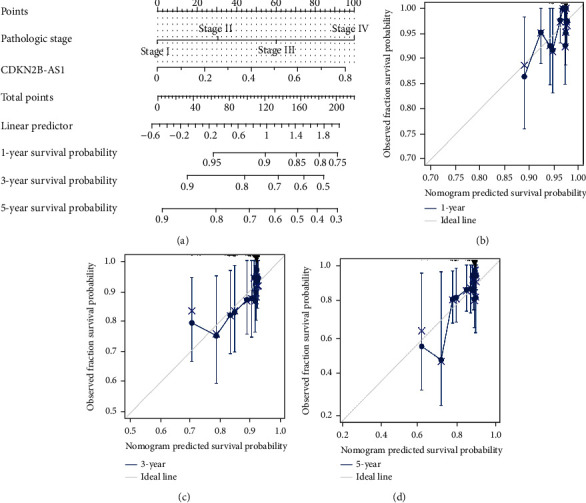
A nomogram based on CDKN2B-AS1 expression and clinical stage. (a) Construction of a nomogram for predicting PFI. Calibration curve of (b) 1 year, (c) 3 years, and (d) 5 years.

To investigate the specific mechanisms by which CDKN2B-AS1 affects the immune microenvironment and tumor progression, we first analyzed the differential expression analysis between the high- and low-CDKN2B-AS1 expressions in the TC cohort (Figures 5(b)–5(d)). Subsequently, the obtained DEGs were analyzed for gene enrichment by three types of algorithms. In KEGG analysis, cytokine-cytokine receptor interaction, hematopoietic cell lineage, and viral protein interaction with cytokine and cytokine receptor were significantly enriched (Figure 5(b)). In GO analysis, cytokine receptor binding, cytokine activity, and receptor ligand activity were mainly enriched (Figure 5(c)). In GSEA analysis based on hallmarks geneset, CDKN2B-AS1 may be involved in the activation or shutdown of many cancer classic pathways, such as epithelial mesenchymal transition, allograft rejection, interferon gamma response, and inflammatory response (Figure 5(d)).

### 3.7. Construction of a Regulatory Network

**Figure 6:**
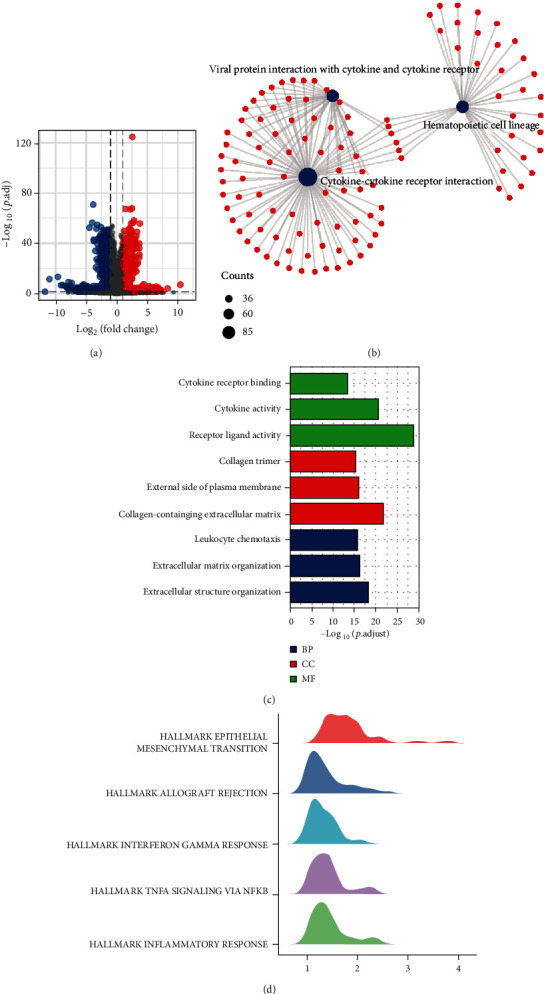
Exploration of the underlying biological mechanisms. (a) Volcanic plot of differential protein-coding genes expression. (b) KEGG analysis. (c) GO analysis. (d) GSEA analysis.

We predicted the target regulators of CDKN2B-AS1 and found that some microRNAs (hsa-let-7d-5p, hsa-miR-4262, hsa-let-7a-5p, hsa-let-7f-5p, hsa-let-7b-5p, hsa-miR-181c-5p, hsa-miR-181d-5p, hsa-let-7i-5p, hsa-miR-98-5p, hsa-miR-181d-5p, and hsa-miR-122-5p, etc.) and two TFs (E2F1 and MYC) may have an effect on the expression of CDKN2B-AS1, as shown in Figure 6.

## 4. Discussion

There is mounting evidence that aberrantly expressed lncRNAs may play a critical role in cancer and developed as oncogenes [16]. Although a growing number of new transcripts have been found, the function of the majority of lncRNAs remains unknown in TC. In this study, the predictive value and underlying biological mechanisms of CDKN2B-AS1 were investigated. We identified that CDKN2B-AS1 was upregulated in TC samples and could be an excellent prognostic indicator. Meanwhile, ssGSEA and ESTIMATE algorithms revealed that CDKN2B-AS1 overexpression may suggest the active state of the immune microenvironment. On the other hand, CDKN2B-AS1 may be involved in the activation or shutdown of many cancer classic pathways, such as epithelial mesenchymal transition, allograft rejection, interferon gamma response, and inflammatory response. Finally, we developed a regulatory network-based CDKN2B-AS1, and two TFs may have an effect on the expression of CDKN2B-AS1.

The long noncoding RNA CDKN2B-AS1 has been linked to a variety of diseases, including cardiovascular illness [17], Alzheimer's disease [18], and type 2 diabetes [19]. The lncRNA CDKN2B-AS1 has been shown in a number of studies to be an independent diagnostic biomarker in breast cancer due to its abnormal expression pattern [20]. Similar findings were found in our investigation, which also indicated CDKN2B-possible AS1's carcinogenic impact in TC. Furthermore, tumor cells, immune cells, and stromal cells may all produce chemokines, which activate various signaling pathways via cell surface receptors to attract immune cell subpopulations to TME, regulating tumor immune responses spatiotemporally [21]. Chemokine networks have the potential to promote or inhibit tumor cell growth, invasion, and metastasis through their effects on tumor cells or tumor-associated immune cells. As a result, targeting chemokines and their receptors is a successful technique for treating cancer. As a consequence, the findings from our DEGs investigation will aid in the development of novel strategies for targeting multichemokine-based therapeutics.

We found that most of the immune cells were upregulated in the CDKN2B-AS1 high expression group, except for Th17 cells, Tgd, pDC, and NK cells. Numerous studies indicate that CD8+ T cell subsets are critical for tumor management, as shown by the link between the quantity of CD8+ T cells in tumors prior to treatment and the response to PD-1 therapy [22]. Circulating CD8+ T cells are stimulated and transformed into effector CD8+ T cells after penetrating tumor tissue [23]. Additionally, CD4+ T helper cells assist DCs in the preparation and activation of CD8+ T cells [24]. Meanwhile, antitumor immunosuppression mediated by CD4+ Treg cells is the primary mechanism of tumor immune evasion and immunotherapy resistance. Due to persistent tumor antigen stimulation, immunosuppressive cells, and physicochemical imbalance, effector CD8+ T cells gradually degenerate, resulting in decreased proliferation and secretion of effector cytokines, a phenomenon referred to as T cell depletion [25]. As a result, the main restriction of tumor immunotherapy is the restoration of CD8+ T cells' anticancer immunological function. Given the effect of CDKN2B-AS on a large number of cells in TME, we may ameliorate this situation by upregulating the expression of CDKN2B-AS1.

However, this study only used the data from the public database TCGA, and there was no condition to detect in vitro, which was a limitation of our study.

## 5. Conclusions

In summary, this study identified CDKN2B-AS1 as a prognostic indicator of TC associated with immune cell infiltration.

## Figures and Tables

**Figure 7:**
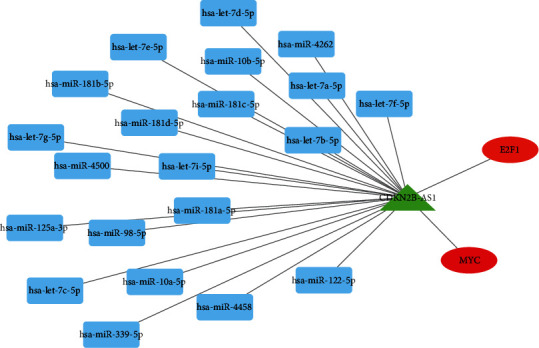
Construction of TFs/microRNAs-CDKN2B-AS1 network. Blue is microRNAs, green is CDKN2B-AS1, and red is TFs.

## Data Availability

The following information was supplied regarding data availability: data is available at the TCGA (https://portal.gdc.cancer.gov/).
